# Cost‐Effective Identification of Hepatocellular Carcinoma from Cirrhosis or Chronic Hepatitis Virus Infection Using Eight Methylated Plasma DNA Markers

**DOI:** 10.1002/advs.202411945

**Published:** 2025-03-26

**Authors:** Tian Yang, Mingda Wang, Nanya Wang, Mingxin Pan, Yu Xu, Qiancheng You, Lanqing Yao, Jiahao Xu, Lihui Gu, Xiaodong Sun, Lei Zhang, Jiayue Xu, Bingsi Li, Guoqiang Wang, Shangli Cai, Guoyue Lv, Feng Shen

**Affiliations:** ^1^ Department of Hepatobiliary Surgery Eastern Hepatobiliary Surgery Hospital Naval Medical University Shanghai 200438 China; ^2^ Department of Hepatobiliary and Pancreatic Surgery General Surgery Center First Hospital of Jilin University Changchun Jilin 130021 China; ^3^ Phase I clinical trials unit First Hospital of Jilin University Changchun Jilin 130021 China; ^4^ Department of Hepatobiliary Surgery II General Surgery Center Zhujiang Hospital Southern Medical University Guangzhou 510280 China; ^5^ Burning Rock Biotech Guangzhou 510300 China

**Keywords:** chronic hepatitis virus infection, early detection, hepatocellular carcinoma, liver cirrhosis, methylated plasma DNA marker

## Abstract

Early detection of hepatocellular carcinoma (HCC) in patients with liver cirrhosis (LC) and/or hepatitis virus B/C infection (HVI) improves survival, highlighting the need for accurate, affordable diagnostic tools. Here, 11 methylated DNA markers (MDMs) are identified during marker discovery. In phase I, each selected MDM is validated in 175 plasma samples (HCC, n = 85; LC/HVI, n = 72) by the CO‐methylation aMplification rEal‐Time PCR (COMET) assay. Of these, 8 MDMs are qualified for phase II study, where a logistic regression model (COMET‐LR) is trained and validated with 336 plasma samples (HCC, n = 211; LC/HVI, n = 113; training vs validation, 2:1). In the validation, the COMET‐LR achieved 90.0% sensitivity at 97.4% specificity. Notably, sensitivity in patients with TNM stage I, diameter<3 cm, AFP‐negative (<20 ng mL^−1^), PIVKA‐II‐negative (<40 mAU mL^−1^) is 82.4%, 77.8%, 88.6%, and 85.7%, respectively. The COMET‐LR outperformed multiple protein markers (AFP, AFP‐L3, and PIVKA‐II) and published scores for HCC screening (GALAD, Doylestown, and ASAP), in terms of both sensitivity and specificity. The assay represents a significant advancement in addressing the unmet need for accurate, non‐invasive, accessible, and cost‐effective early detection tools for LC/HVI individuals. Further validation in a prospective cohort is warranted.

## Graphical abstract

Early detection of hepatocellular carcinoma in patients with liver cirrhosis and/or hepatitis virus B/C infection improves survival. Through marker discovery, 8 methylated DNA markers are identified and a logistic regression model (COMET‐LR) is built in the training set, which achieved 90.0% sensitivity at 97.4% specificity in the validation set. This model outperformed protein markers (AFP, AFP‐L3%, and PIVKA‐II) and published scores (Doylestown, GALAD, and ASAP).

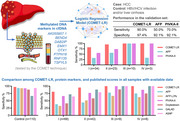

## 1 Introduction

Hepatocellular carcinoma (HCC) stands as a critical global health concern, particularly in individuals afflicted with liver cirrhosis (LC) and/or hepatitis virus B/C infections (HVI).^[^ ^1^ ^]^ The imperative for early detection is paramount, given that interventions at advanced stages often yield limited efficacy.^[^ ^2^ ^]^ Existing diagnostic modalities, including alpha‐fetoprotein (AFP) and ultrasound, though widely utilized, are fraught with limitations, particularly in terms of accuracy.^[^ ^3^, ^4^ ^]^

Plasma‐based testing, with fewer requirements for medical resources compared to radiological assessments, would be ideal for HCC surveillance in the LC/HVI population. Plasma cell‐free DNA (cfDNA) carries the signals produced by HCC.^[^ ^5^ ^]^ Compared with other genomic alterations (such as mutations, small insertions/deletions, and copy number variations), the alteration of DNA methylation appears earlier and is more abundant in cfDNA.^[^ ^6^ ^]^ Moreover, in a multi‐cancer early detection study, targeted methylation assays displayed a better clinical limit of detection (defined as the circulating tumor allele fraction at which the probability of detecting a cancer signal was at least 50% while maintaining a 98% specificity) in comparison with targeted sequencing of small somatic variants and low‐pass whole‐genome sequencing,^[^ ^7^ ^]^ standing out as a promising avenue for the early detection of HCC.

In contrast with using large panels to detect cfDNA methylation,^[^ ^8^ ^]^ the quantitative methylation‐specific polymerase chain reaction (qMSP) technique incurs lower costs,^[^ ^9^, ^10^, ^11^, ^12^, ^13^ ^]^ rendering it suitable for widespread application especially in resource‐constrained settings. Leveraging the qMSP‐based technique, Exact Science developed a multitarget HCC blood test including two methylated DNA markers (MDMs; *HOXA1*, and *TSPYL5*), AFP, and sex.^[^ ^10^, ^12^, ^13^ ^]^ This assay yielded a sensitivity of 82.1% in Barcelona Clinic Liver Cancer (BCLC) stage 0–A cases at a specificity of 86.9% in LC/HVI controls in the clinical validation,^[^ ^13^ ^]^ and there remains significant potential for further improvement in performance.

In this study, we developed a cost‐effective assay utilizing qMSP to assess the methylation levels of eight specific genes associated with HCC. Our assay leverages the power of cfDNA methylation analysis to provide a sensitive and specific tool for identifying individuals at an early stage of HCC, enabling timely interventions, and potentially improving patient outcomes.

## 2 Results

### 2.1 Marker Discovery

**Figure 1:**
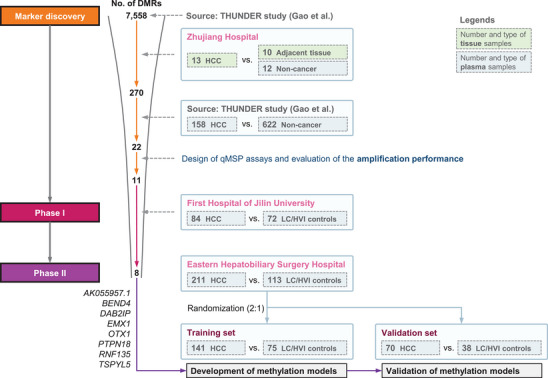
Schematic diagram of study design. Abbreviations: HCC = hepatocellular carcinoma, HVI = hepatitis virus infection, LC = liver cirrhosis.

The schematic diagram of this study is shown in **Figure** **1**. In our previous THUNDER study aiming to develop a multi‐cancer detection test for cancers in colorectum, esophagus, liver, lung, ovary, and pancreas, we distinguished a total of 161984 CpG sites (7558 methylation regions) for cancer detection and localization from the methylation data of cancer/normal tissues (The Cancer Genome Atlas [TCGA] database) and white blood cells (GSE40279).^[^ ^14^ ^]^ Here, to locate the most relevant regions for HCC from these CpG sites, we first compared their methylation levels in 13 HCC tissues, 10 adjacent tissues, and 12 plasma samples from non‐cancer participants with LC/HVI (characteristics, see Table S1, Supporting Information) and identified ≈270 differentially methylated regions (DMRs) using the following criteria: 1) sequencing depth >100; 2) delta value >0.10 between tumor and adjacent tissue as well as tumor and plasma samples. Then, we further narrowed down the DMRs of interest by contrasting their methylation levels in the cfDNA samples from 622 healthy donors and 158 HCC patients (characteristics, see Table S2, Supporting Information). In total, 22 hypermethylated DMRs were selected following the criteria: 1) sequencing depth >100; 2) delta value >0.01 between HCC and healthy cfDNA samples; 3) >8‐fold change; 4) Wilcoxon test, FDR<0.05. We designed qMSP assays for these 22 DMRs and half of them were abandoned due to poor amplification performance (low specificity and/or amplification efficiency). The remaining 11 MDMs (*AK055957.1*, *BEND4*, *DAB2IP*, *EMX1*, *OTX1*, *PTPN18*, *RNF135*, *SEPTIN9*, *SOCS1*, *TSPYL5*, and *VIM*) were chosen as candidate MDMs for further validation in the Phase I study. These MDM‐associated genes are known to play crucial roles in tumorigenesis, specifically cell differentiation and proliferation, metabolism, cytokine signaling, and epithelial‐mesenchymal transition (summarized in Table S4, Supporting Information).^[^ ^11^, ^12^, ^15^ ^]^ Further validation was implemented in The Cancer Genome Atlas‐Liver Hepatocellular Carcinoma (TCGA‐LIHC) cohort where methylation levels of HCC tissues were assessed by the HM450 microarray.^[^ ^16^ ^]^ The probes closest to the 11 identified MDMs were analyzed. All these probes had a higher methylation level in the HCCs compared to adjacent tissues (Figure S1, Supporting Information), independent of HBV/HCV status (Figure S2, Supporting Information). This indicates that these 11 MDMs may be applicable for both viral and non‐viral subjects (Figure 1).

### 2.2 Phase I Pilot Study

**Figure 2:**
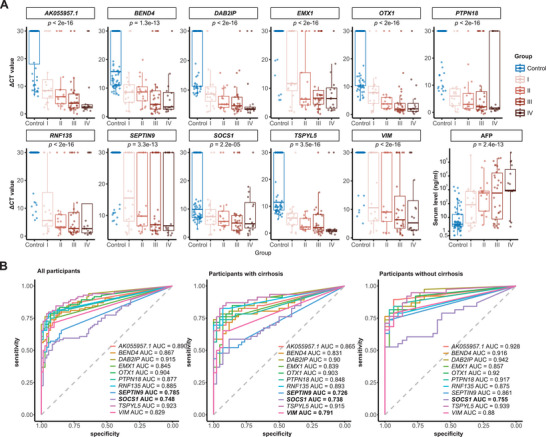
Phase I pilot study. A) The methylation levels of 11 MDMs and AFP concentration in LC/HVI controls and HCC cases (the Mann–Whitney test; sample size: stage I [n = 20], stage II [n = 17], stage III [n = 31], stage IV [n = 16], control [n = 72]; data were presented with median and interquartile range). B) Receiver operator characteristic curves of 11 MDMs in all participants (n = 156, left panel), cirrhotic participants (n = 88, middle panel), and non‐cirrhotic participants (n = 68, right panel). Abbreviations: AFP = alpha fetoprotein, AUC = area under the receiver operator characteristic curve, Ct = threshold cycle, HCC = hepatocellular carcinoma, HVI = hepatitis virus infection, LC = liver cirrhosis, MDM = methylated DNA marker.

The pilot study included 84 HCC cases and 72 LC/HVI controls from the First Hospital of Jilin University (characteristics, see **Table** **1**; Table S5, Supporting Information). Most subjects were male (84.7%) and HBV‐infected (90.5%) and about half of the participants had liver cirrhosis (54.8%). Age, sex, HBV/HCV infection, and cirrhotic status were comparable between cases and controls (Table 1). The 11 hypermethylated MDMs exhibited a remarkable increase in methylation level (reflected by a decrease of ∆cycle threshold [Ct]Barcelona Clinic Liver Cancer) in cancer cases compared to LC/HVI controls (p < 1.9 × 10^−5^, **Figure** **2**A), and their methylation levels trended to be positively correlated with staging (Figure 2A). In contrast to MDMs, AFP showed poorer power to differentiate between cancer cases and LC/HVI (p = 0.0029, Figure 2A).

**Table 1:** Clinical features of the cases and controls in phase I and II studies.

				Phase II					
	Phase I			Training set			Validation set		
	HCC cases (n = 84)	LC/HVI controls (n = 72)	p value	HCC cases (n = 141)	LC/HVI controls (n = 75)	p value	HCC cases (n = 70)	LC/HVI controls (n = 38)	p value
Age	56 (48–63)	54 (49–58)	0.15	58 (50–63)	57 (52–62)	0.88	55 (50–63)	58 (52–62)	0.43
Sex (male)	61 (84.7%)	75 (88.2%)	0.70	111 (78.7%)	57 (76.0%)	0.78	57 (77.0%)	30 (78.9%)	0.89
HBV (positive)	76 (90.5%)	66 (91.7%)	1.00	120 (85.1%)	67 (89.3%)	0.51	65 (87.8%)	35 (92.1%)	0.64
HCV (positive)	2 (2.4%)	3 (4.2%)	0.86	5 (3.5%)	4 (5.3%)	0.79	5 (6.8%)	1 (2.6%)	0.59
Cirrhosis	46 (54.8%)	42 (58.3%)	0.77	46 (32.6%)	32 (42.7%)	0.19	23 (31.1%)	19 (50.0%)	0.091
AFP (ng/ml)	663.2 (9.8–3056.4)	3.3 (2.3–16.8)	0.007	39.7 (4.6–406.4)	3.4 (2.5–4.8)	0.032	19.1 (4.3–358)	3.4 (2.1–4.9)	0.13
AFP‐L3%	NA	NA		8.1 (0.9–17.8)	0.9 (0.9–0.9)	<0.001	2.6 (0.9–19.2)	0.9 (0.9–0.9)	<0.001
PIVKA‐II (mAU/ml)	NA	NA		307 (53–2574)	9.9 (7.0–15.5)	0.002	308 (28.3–2428)	8.3 (6.1–11.5)	0.013
TNM stage									
I	20 (23.8%)			67 (47.5%)			34 (48.6%)		
II	17 (20.2%)			47 (33.3%)			23 (32.9%)		
III	31 (36.9%)			20 (14.2%)			10 (14.3%)		
IV	16 (19.0%)			7 (5.0%)			3 (4.3%)		
Diameter of the largest lesion (cm)									
	9.7 (4.4–13.0)			5.0 (2.8–6.2)			4.2 (3.1–7.6)		
Tumor number, n (%)									
1	29 (34.5%)			102 (72.3%)			51 (72.9%)		
2–3	21 (25.0%)			25 (17.8%)			11 (15.7%)		
>=4	34 (40.5%)			14 (9.9%)			8 (11.4%)		

As shown in Figure 2B, the ROC curve for MDMs first rose rapidly from the lower left corner to reach a status of high specificities (>90%) and moderate sensitivities (50%–75%) and then gradually converged to the upper right corner. This characterization suggests that the strength of these MDMs may be high specificity rather than high sensitivity.

In general, early detection tools for HCC are less specific in cirrhotic controls than those without cirrhosis.^[^ ^10^, ^17^ ^]^ To ensure that our final model could perform well in both the cirrhotic and non‐cirrhotic population, we set the following criterion for filtering MDMs, i.e., an area under the receiver operator characteristic curve (AUC) greater than 0.80 in both cirrhotic and non‐cirrhotic subgroups (HCCs vs LC/HVI controls). As shown in Figure 2B, *SEPTIN9*, *SOCS1*, and *VIM* had an AUC of 0.726, 0.738, and 0.791 in the cirrhotic subgroup, respectively; *SOCS1* also performed poorly in the non‐cirrhotic subgroup (AUC = 0.755). Given this, *SEPTIN9*, *SOCS1*, and *VIM* were excluded from evaluation in the following phase II study.

### 2.3 Phase II Clinical Validation

The phase II study included 223 cases (HCC, n = 211) and 113 LC/HVI controls from the Eastern Hepatobiliary Surgery Hospital (characteristics, see Table 1; Table S5, Supporting Information). Similar to the phase I study, most subjects were male (77.5%) and HBV‐infected (87.3%). Cirrhosis was observed in 36.7% of participants. To better assess the potential value of MDMs in early detection, we collected a large proportion of patients with early‐stage cancer (stage I: 47.9%; stage II: 33.2%). All subjects were stratified and randomly allocated into the training set and the validation set at a 2:1 ratio, considering age, gender, cancer subtype, staging, and cirrhotic status. Clinical characteristics were comparable between cases and controls in both the training and the validation sets (Table 1; Table S5, Supporting Information).

In the training set, we attempted two approaches to developing models based on these 8 MDMs. The first one was modeling with logistic regression, referred to as the “COMET‐LR” (coefficient for each MDM, see Table S6, Supporting Information). The second one served as a control with limited risk of overfitting, which was simply summing the delta threshold cycle (∆Ct) values of the 8 MDMs, referred to as the “COMET‐SumΔCt”. The AUC was 0.943 and 0.937 for the COMET‐LR and the COMET‐SumΔCt, respectively.

**Figure 3:**
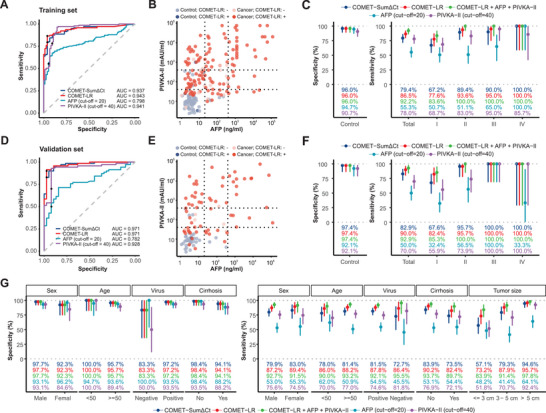
Phase II clinical validation. A) Receiver operator characteristic curves of the COMET‐Sum∆Ct, the COMET‐LR, AFP, and PIVKA‐II in the training set (n = 216). B) The levels of AFP and PIVKA‐II in samples grouped by the COMET‐LR results in the training set (n = 216). C) Sensitivity (n‐141) and specificity (n = 75) of the COMET‐Sum∆Ct, the COMET‐LR, the COMET‐LR+AFP+PIVKA‐II, AFP, and PIVKA‐II in the training set (the Clopper‐Pearson method). D) Receiver operator characteristic curves of the COMET‐Sum∆Ct, the COMET‐LR, AFP, and PIVKA‐II in the validation set (n = 108). E) The levels of AFP and PIVKA‐II in samples grouped by the COMET‐LR results in the validation set (n = 108). F) Sensitivities (n = 70) and specificities (n = 38) of the COMET‐Sum∆Ct, the COMET‐LR, the COMET‐LR+AFP+PIVKA‐II, AFP, and PIVKA‐II in the validation set (the Clopper–Pearson method). G) Subgroup analysis of sensitivity (n = 70) and specificity (n = 38) in all samples included in the phase II study (the Clopper‐Pearson method). Abbreviations: AFP = alpha fetoprotein, AUC = area under the receiver operator characteristic curve, cHCC‐CC = combined hepatocellular‐cholangiocarcinoma, COMET = CO‐methylation aMplification rEal‐Time polymerase chain reaction, Ct = threshold cycle, HBV = hepatitis B virus, HCV = hepatitis C virus, LR = logistic regression, MDM = methylated DNA marker, NA = not applicable, PIVKA‐II = protein induced by vitamin K absence II.

As mentioned above, these 8 MDMs were strong in high specificity instead of high sensitivity. Based on this, we determined cut‐off values for the two models (COMET‐LR: 0.625; COMET‐SumΔCt: 169.45) that led to a high specificity of 96.0% (95% confidence interval [CI]: 88.8%‐99.2%). Under these cut‐offs, the COMET‐LR and the COMET‐SumΔCt yielded sensitivities of 86.5% (95% CI: 79.8%–91.7%) and 79.4% (95% CI: 71.8%–85.8%), respectively (**Figure** **3**A). With commonly used cut‐off values for AFP (20 ng/mL) and protein induced by vitamin K absence II (PIVKA‐II, 40 mAU/mL) in clinical settings,^[^ ^18^ ^]^ the COMET‐LR identified 82.5% of AFP‐negative cancer cases and 74.2% of PIVKA‐II‐negative cancer cases (Figure 3B).

To further use AFP and PIVKA‐II to improve the sensitivity of the COMET‐LR while ensuring high specificity, we designed a combination model (referred to as “COMET‐LR+AFP+PIVKA‐II”) where positivity was determined by satisfying any of the following conditions: i) COMET‐LR score ≥ 0.625, ii) AFP ≥ 20 ng mL^−1^ and PIVKA‐II ≥ 40 mAU mL^−1^, iii) AFP ≥ 400 ng mL^−1^, and iv) PIVKA‐II ≥ 400 mAU mL^−1^. The combination model increased the sensitivity to 92.2% (95% CI: 86.5%–96.0%) while maintaining no reduction in specificity (Figure 3C). Specifically, the COMET‐LR and the combination model achieved a favorable sensitivity of 77.6% and 83.6% in stage I cancer cases, superior to AFP (50.7%) and PIVKA‐II alone (68.7%, Figure 3C).

In the validation set, the AUC was 0.971 for both the COMET‐LR and the COMET‐SumΔCt, higher than that of AFP (0.782) and PIVKA‐II (0.928, Figure 3D). The COMET‐LR identified 88.6% of AFP‐negative cancer cases and 85.7% of PIVKA‐II‐negative cancer cases (Figure 3E). At pre‐specific cut‐offs, the COMET‐LR and the COMET‐SumΔCt yielded sensitivities of 90.0% (95% CI: 80.5%–95.9%) and 82.9% (95% CI: 72.0%–90.8%) at specificity of 97.4% (95% CI: 86.2%–99.9%), which was better than the performance of AFP (sensitivity: 50.0%; specificity: 92.1%) and PIVKA‐II (sensitivity: 70.0%; specificity: 92.1%; Figure 3F). Based on the COMET‐LR, incorporation of AFP and PIVKA‐II slightly increased the sensitivity to 92.9% (Figure 3F). In terms of early detection, the COMET‐LR and the combination model reached a remarkable sensitivity of 82.4% and 85.3% in stage I cancer cases, which outperformed AFP (32.4%) and PIVKA‐II alone (55.9%, Figure 3F).

Since the consistent performances of methylation models in the training and the validation sets, subgroup analysis was performed in all samples rather than only in those included in the validation set, for greater statistical power. The sensitivity and specificity of the methylation models built in the training set were similar between subgroups defined by sex, age, HBV/HCV infection, and cirrhotic status (Figure 3G). It is important to note that in our samples, the subgroup with non‐viral infections has a relatively small sample size, so the interpretation of results for this group should be done with caution. The sensitivities of methylation models grew with increasing tumor size (Figure 3G). Of note, in the HCC samples, the COMET‐LR yielded sensitivities of 67.6% and 86.5% in BCLC stage 0 and A cases, respectively (Figure S3A, Supporting Information).

Collectively, the COMET‐LR constructed based on the selected 8 MDMs achieved high sensitivity and specificity in both the training and the validation sets, and its performance was consistent in different subgroups defined by sex, age, HBV/HCV infection, and cirrhotic status.

### 2.4 Comparison between MDM‐based Models and Published Scores for HCC Early Detection

We sought to further compare MDM‐based models with published scores (gender, age, Lens culinaris agglutinin‐reactive AFP, AFP, and des‐g‐carboxy‐pro‐thrombin score [GALAD], age, sex, AFP, and PIVKA‐II [ASAP], and Doylestown) for HCC early detection. In the phase II study, 238 (74.1%) had available data on Lens culinaris agglutinin A‐reactive fraction of AFP (AFP‐L3) which is required for the GALAD score. Of these 238 participants, age, sex, and HBV/HCV infection were comparable between cases and controls (P > 0.20), while controls had a higher rate of cirrhosis compared to cases (44.5% vs 28.1%, P = 0.012; Table S7, Supporting Information), which might lead to a slight underestimation of specificity.

**Figure 4:**
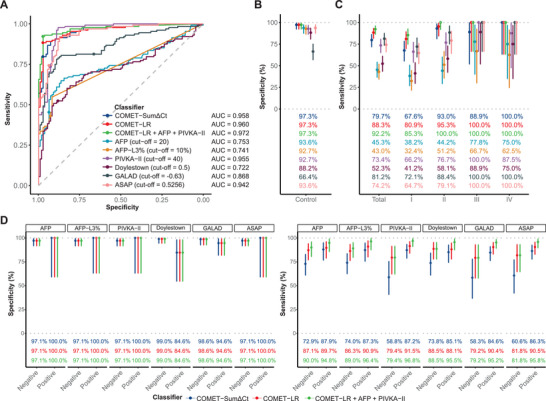
Comparison between MDM‐based models and published scores for HCC early detection. A) Receiver operator characteristic curves of methylation models, protein markers, and published scores (n = 238, the Clopper‐Pearson method). B,C) Sensitivities (n = 128, B) and specificities (n = 110, C) of methylation models, protein markers, and published scores (the Clopper–Pearson method). D) Specificities (n = 128) and sensitivities (n = 110) of methylation models in subgroups defined by protein markers and published scores (the Clopper‐Pearson method). Abbreviations: AFP = alpha fetoprotein, ASAP = age, sex, AFP, and PIVKA‐II, AUC = area under the receiver operator characteristic curve, cHCC‐CC = combined hepatocellular‐cholangiocarcinoma, COMET = CO‐methylation aMplification rEal‐Time polymerase chain reaction, Doylestown = age, sex, AFP, alkaline phosphatase, and alanine aminotransferase, GALAD = gender, age, Lens culinaris agglutinin‐reactive AFP, AFP, and des‐g‐carboxy‐pro‐thrombin, HBV = hepatitis B virus, HCV = hepatitis C virus, LR = logistic regression, NA = not applicable, PIVKA‐II = protein induced by vitamin K absence II.

The AUC values of the COMET‐SumΔCT, the COMET‐LR, and the COMET‐LR+AFP+PIVKA‐II were 0.958, 0.960, and 0.972, respectively, surpassing protein markers (AFP: 0.753; AFP‐L3: 0.741; PIVKA‐II: 0.955) and published scores (GALAD: 0.868; ASAP: 0.942; Doylestown: 0.722; **Figure** **4**A). Interestingly, the AUC for PIVKA‐II in our study (0.955) was higher compared to previous reports in HBV‐infected (0.899),^[^ ^19^ ^]^ HCV‐infected (0.859),^[^ ^17^ ^]^ and nonalcoholic steatohepatitis subsets (0.87).^[^ ^20^ ^]^ Of note, the COMET‐LR yielded a sensitivity of 88.3% at a high specificity of 97.3%, both of which were superior to protein markers and those of scores at the published cut‐off values (GALAD: –0.63; ASAP: 0.5256; Doylestown: 0.5; Figure 4B,C).^[^ ^19^, ^21^, ^22^ ^]^ The advantage of the COMET‐LR over published scores was mainly in the early‐stage cases (Figure 4C; Figure S3B, Supporting Information). The COMET‐LR reached sensitivities of 80.9% and 95.3% in stage I and II cases, respectively, superior to GALAD (72.1% and 88.4%), ASAP (64.7% and 79.1%), and Doylestown (41.2% and 58.1%; Figure 4C).

When the cut‐off values of protein markers and published scores were adjusted to achieve the same specificity as the COMET‐LR (97.3%), their sensitivities in stage I cases remarkably dropped (AFP: 23.5%; AFP‐L3: 30.9%; PIVKA‐II: 30.9%; Doylestown: 25.0%; GALAD: 42.6%; ASAP: 39.7%), which were inferior to the COMET‐LR (80.9%; Figure S4, Supporting Information). This advantage remained when cut‐off values were adjusted to yield a specificity of 94.5% (Figure S5, Supporting Information).

Moreover, in cases and controls misclassified by protein markers and published scores, the COMET‐LR showed high accuracy. Taking GALAD for an example, the COMET‐LR had a specificity of 94.6% in GALAD‐positive controls (Figure 4D) and a sensitivity of 79.2% in GALAD‐negative cases (Figure 4D). The sample size in certain subgroups was not sufficient to draw a strong conclusion, and thereby these results need more data to verify. Collectively, the MDM‐based models showed advantages over protein markers and published scores in the early detection of HCC in LC/HVI population.

### 2.5 Exploratory Analysis in cHCC‐CC and ICC

Since HVI and LC are risk factors for both intrahepatic cholangiocarcinoma (ICC) and combined HCC‐cholangiocarcinoma (cHCC‐CC),^[^ ^23^ ^]^ it might be more useful if an early detection tool for HCC could also detect ICC and cHCC‐CC. Here, in a small dataset (TCGA‐CHOL: ICC, n = 36; adjacent, n = 15), we found that 5 out of 8 MDMs (except *DAB2IP*, *PTPN18*, and *RNF135*) included in the COMET‐LR had a remarkably higher methylation level in ICCs compared to adjacent tissues (Figure S6, Supporting Information), which might be preliminary due to the small sample size.

In plasma samples, we further compared the methylation levels of these MDMs among ICCs, cHCC‐CCs, HCCs, and controls (Figure S7, Supporting Information; characteristics, see Table S3, Supporting Information; Table 1). Of note, all ICC and cHCC‐CC samples were from stage I‐II patients. The methylation levels of all 8 MDMs were higher in ICCs and cHCC‐CCs compared to controls. Among them, the methylation levels in cHCC‐CCs were very similar to those in HCCs. However, in ICCs, the methylation levels of ***DAB2IP*, *PTPN18***, and ***RNF135*** were intermediate between those of HCCs and controls, which is consistent with the findings from the TCGA‐CHOL dataset (Figure S6, Supporting Information). Despite the imperfect performance of these 3 MDMs in ICCs, the COMET‐LR model achieved high sensitivity in early‐stage ICCs (90.0%) and cHCC‐CCs (100.0%), greater than AFP (ICC: 5.0%; cHCC‐CC: 61.5%) and PIVKA‐II (ICC: 20.0%; cHCC‐CC: 92.3%; Figure S8, Supporting Information).

## 3 Discussion

We reported 8 MDMs that, when detected from cfDNA using our COMET technique, can distinguish HCC cases from LV/HVI controls with high sensitivity and specificity. Leveraging the insight from our previous THUNDER study and the methylome data of independent tissues and plasma samples, we identified 11 HCC‐specific MDMs. In phase I, these 11 MDMs were downsized to 8 that were used for developing models in phase II, where early‐stage HCCs were enriched to reflect the intended surveillance population. In the validation set of the phase II study, the COMET‐LR model achieved 90.0% sensitivity at 97.4% specificity and its sensitivity in patients with TNM stage I, diameter <3 cm, AFP‐negative (<20 ng mL^−1^), PIVKA‐II‐negative (<40 mAU mL^−1^) was 82.4%, 77.8%, 88.6%, and 85.7%, respectively. In addition, the COMET‐LR can detect 90.0% and 100.0% of early‐stage ICCs and cHCC‐CCs, respectively.

A high early‐stage sensitivity can increase the opportunity for curative treatment. The current diagnostic modality used in high‐risk individuals is AFP plus ultrasound, which only distinguished 63% of early‐stage HCCs at a specificity of ≈84% reported in a recent meta‐analysis.^[^ ^3^ ^]^ In clinical validation, the COMET‐LR achieved high sensitivities of 82.4% and 95.7% in stage I and II cancer cases, respectively, at a high specificity of 97.4%. Although the AUC of PIVKA‐II in our study (0.955) was notably higher compared to previous reports in HBV‐infected (0.899),^[^ ^19^ ^]^ HCV‐infected (0.859),^[^ ^17^ ^]^ and nonalcoholic steatohepatitis subsets (0.87),^[^ ^20^ ^]^ the COMET‐LR outperformed PIVKA‐II and ASAP in specificity (97.3% vs 92.7% and 93.6%) and sensitivity in stage I cases (80.9% vs 66.2% and 64.7%). In addition, when the cut‐off values of all models and protein markers were adjusted to achieve the specificity of the COMET‐LR (97.3%), their sensitivities in stage I cases were below 42.6%, remarkably lower than the 80.9% of the COMET‐LR. These findings demonstrated that the COMET‐LR was superior to multiple protein markers (AFP, AFP‐L3, and PIVKA‐II) and published scores (GALAD, Doylestown, and ASAP) in terms of both early‐stage sensitivity and specificity.

The use of qMSP‐based techniques in identifying early‐stage HCC cases from high‐risk controls has been evaluated by several groups (summarized in Table S8, Supporting Information).^[^ ^9^, ^10^, ^11^, ^12^, ^13^ ^]^ Of these, Exact Science achieved the optimal results from rigorously designed studies, ranging from marker elimination,^[^ ^10^ ^]^ marker selection,^[^ ^12^ ^]^ to algorithm development and clinical validation.^[^ ^13^ ^]^ In their clinical validation, the multitarget HCC blood test (mt‐HBT) consisting of two MDMs (*HOXA1* and *TSPYL5*), AFP, and sex yielded a sensitivity of 82.1% in BCLC stage 0–A cases at a specificity of 86.9% in LC/HVI controls.^[^ ^13^ ^]^ Herein, compared to the mt‐HBT, our COMET‐LR achieved a comparable sensitivity of 84.8% at a higher specificity of 97.4% in the validation set.

The performance of our COMET‐LR model was consistent in different subgroups defined by sex, age, HBV/HCV infection, and cirrhotic status, indicating the potential for benefiting more at‐risk populations. However, it is important to note that our study was conducted in China, where HBV infection is the predominant etiology of HCC. This inherent bias may have affected the generalizability of our findings, particularly for patients with HCV infection or non‐viral cirrhosis, such as those resulting from non‐alcoholic fatty liver disease (NAFLD). The smaller sample size for these subgroups further limits the statistical power to draw definitive conclusions. Despite these limitations, our analysis of the TCGA dataset demonstrated that the MDMs included in the COMET models were consistently hypermethylated across HBV‐infected, HCV‐infected, and non‐viral HCCs. This suggests that the assay has broader applicability and potential utility for patients with HCV infection or non‐viral cirrhosis. Future studies with larger and more diverse cohorts, particularly those focusing on underrepresented etiologies like HCV and NAFLD‐related HCC, will be essential to validate and further refine the utility of this approach. In addition, the COMET‐LR can also efficiently detect early‐stage ICC and cHCC‐CC samples in our study, although the sample size in this subset is small and may not be adequately studied within the context of this research. Taken together, the identified MDMs and the COMET model may be applicable to all subsets of primary liver cancers.

In our study, cases and controls were matched for age and sex in our phase II study, and neither age nor sex was included for modeling. This approach may contribute to the consistent performance of the COMET‐LR model across different sexes and ages. While older age and male sex are linked to an increased risk of HCC,^[^ ^24^ ^]^ when designing diagnostic aids rather than tools for risk assessment, it may be more appropriate to incorporate factors related to the molecular characteristics associated with HCC. When age and sex are included in the model, it may be more likely to detect HCC in elderly males at the expense of performance in the younger female population. For example, from the results of the mt‐HBT (including MDMs, AFP, and sex) in clinical validation, its early‐stage sensitivity was 83% at 84% specificity in men, while the sensitivity was lower at 77% with a higher specificity at 91% in women.^[^ ^13^ ^]^ Early detection of HCC is equally critical regardless of age or sex, however, the variation in model performance across different ages and sexes may pose challenges in meeting the clinical needs of younger females.

As for limitations, first, the retrospective design limited the generalizability of our findings. While we used samples from three independent centers respectively for marker discovery, phase I, and phase II, to potentially enhance credibility and validity, further assessment of our findings in a prospective cohort comprising high‐risk individuals is warranted. Second, as mentioned above, participants with HCV infection or non‐viral cirrhosis were insufficient in our study. The results of subgroup analysis based on these factors should be interpreted with caution. Third, a subset of subjects lacked information on AFP‐L3% in the phase II study, and thereby the comparison of methylation models with AFP‐L3% and GALAD is suboptimal. Moreover, fucosylated kininogen was not detected in all samples to the extent that it was impossible to compare methylation models with Doylestown Plus.^[^ ^25^ ^]^ Fourth, previous early detection studies have found that early detection models based on DNA methylation were associated with cancer prognosis.^[^ ^26^ ^]^ Currently, the follow‐up time for patients included in our study is relatively short, and the recurrence events are not yet sufficiently mature for related analyses.

As a blood‐based test, our COMET assay offers advantages in terms of reduced human resource requirements and higher accuracy for early HCC detection compared to abdominal ultrasound plus AFP. A modeling study on HCC surveillance has highlighted a preference for blood‐based tests over ultrasound plus AFP due to increased convenience and shorter time commitment required.^[^ ^27^ ^]^ Moreover, the economic feasibility of our assay is a critical aspect, addressing a significant gap in the current landscape of plasma‐based detection approaches. The cost‐effectiveness of our assay enhances its potential for widespread adoption, particularly in settings where financial constraints may limit access to more expensive assays, such as those detecting cfDNA fragmentomics or ctDNA mutations. However, compared with protein markers (e.g., AFP and PIVKA‐II) and the published scores based on these markers (e.g., ASAP), whether the socioeconomic benefit from the superior performance of COMET‐LR would cover its relatively higher cost warrants further investigations. The data from case‐control studies are not well‐suited for conducting health economic analyses. We are currently undertaking a prospective cohort study, and we plan to use the results of this study to analyze the cost‐effectiveness of the COMET‐LR model. In addition, multi‐dimensional interactive cascading nanochips have been explored for their promising performance in the early detection of HCC. The combination of the methylation model and nanochip technology may have potential for further research.^[^ ^28^ ^]^

In summary, our research presents a promising qPCR‐based assay for the early detection of HCC in LC/HVI patients, demonstrating superior sensitivity and specificity compared to multiple protein markers and published scores. By harnessing the power of cfDNA methylation analysis and coupling it with the advantages of a low‐cost qPCR platform, our assay represents a significant advancement in addressing the unmet need for accurate, non‐invasive, accessible, and cost‐effective early detection tools for individuals at high risk of developing HCC. Further investigation in a large prospective cohort study is warranted to evaluate its clinical utility.

## 4 Experimental Section

### Study Design and Participants

This study includes three parts: i) marker discovery, ii) phase I pilot study, and iii) phase II clinical validation. This study followed the principles of the Declaration of Helsinki and was approved by the Ethics Committee or Institution Review Board of all included centers (2021‐KY‐013‐01 [Zhujiang Hospital], 23K012‐001 [First Hospital of Jilin University], and EHBHKY2020‐01‐093 [Eastern Hepatobiliary Surgery Hospital]). This report followed the Strengthening the Reporting of Observational Studies in Epidemiology (STROBE) reporting guideline. Written informed consent from all participants was obtained.

In marker discovery, 13 HCC tissues, 10 adjacent tissues, and 12 plasma samples from non‐cancer participants were collected from Zhujiang Hospital to identify HCC‐related MDMs (clinical features, see Table S1, Supporting Information). These samples were sequenced by the enhanced linear‐splinter amplification sequencing (ELSA‐seq) technique (coverage >1000x) as previously described,^[^ ^29^ ^]^ using a customized panel including 161984 CpG sites (7558 methylation regions).^[^ ^14^ ^]^ This panel had been designed for multi‐cancer early detection described in the previous study (THUNDER).^[^ ^14^ ^]^ Then, the identified MDMs were further narrowed down by comparing the methylation data of 622 healthy donors and 158 HCC patients, which were retrieved from the THUNDER study (clinical features, see Table S2, Supporting Information).^[^ ^14^ ^]^ Probes were designed for the final selected MDMs for qMSP assays. MDMs with better amplification performance were entered into the subsequent phase I study. Addition validation was implemented in the TCGA‐LIHC dataset, where methylation levels were assessed by Infinium HumanMethylation450 (HM450) BeadChip.^[^ ^16^ ^]^

Participants for phase I were identified in First Hospital of Jilin University, and those for phase II and exploratory analysis were included from Eastern Hepatobiliary Surgery Hospital (clinical features, see Table 1; Table S3, Supporting Information). In phase I and II, key eligibility criteria for cases included an age range of 18 to 75 years, a clinical diagnosis of HCC, and concomitant LC and/or HVI. Those with cHCC‐CC or ICC were included for exploratory analysis. The plasma samples of cancer patients collected before any anti‐cancer treatment and within 6 months before diagnosis were tested with qMSP. As for controls, patients with LC/HVI undergoing surveillance without evidence of HCC and cHCC‐CC were included. The plasma samples of controls collected within 6 months before the latest follow‐up were used for this study.

### CO‐methylation Amplification Real‐Time PCR (COMET) Assay

The qMSP primers, probes, and blockers were designed using the Primer Express 3.0.1 (Thermo‐Fisher Scientific, 4363991). Plasma cfDNA was extracted from 4 mL plasma with the QIAamp Circulating Nucleic Acid Kit (Qiagen, 55114). Around 15–40 ng of the extracted cfDNA was subjected to bisulfite conversion using the EZ‐96 DNA methylation‐lightning MagPrep (Zymo Research, D5047) according to the manufacturer's protocol.

Pre‐amplification was performed in the mixture of 10.5 µL of bisulfite‐converted cfDNA with 2 µL of the MSP primer mix and 12.5 µL of the Entrans 2X qPCR Probe Master Mix (Abclonal, RK21208). The pre‐amplification started with initial denaturation (95 °C, 5 min) which was followed by 8 cycles of denaturation (95 °C, 15 s) and annealing/extension (60 °C, 30 s). Then, 4 µL of the pre‐amplified DNA product, 10 µL of Entrans 2X qPCR Probe Master Mix (Abclonal, RK21208) and 6 µL of the qMSP pre‐mix (primers, probes, and blockers) were mixed for qPCR, including two steps: i) initial denaturation (95 °C, 10 min) and ii) 45 cycles of denaturation (95 °C, 15 s) and annealing/extension (60 °C, 30 s).

The methylation level of each MDM was normalized by a non‐methylated internal control, *ACTB*, whose Ct value was ≈20 in the present study. The valid methylation percentage of each marker was defined by ∆Ct by subtracting the Ct value of *ACTB* from that of each MDM. When the Ct value of MDMs reached the upper limit (i.e., 45), which indicated that its true Ct value may be largely greater than 45, its ∆Ct value will be set directly at 30 in this study.

### Estimation of Serum Protein Markers

As for AFP, AFP‐L3, and PIVKA‐II, peripheral blood was collected before treatments and separated by centrifugation (700 *g*, 10 min). The serum was aliquoted and immediately frozen at −80 °C until testing. The sample storage facilities and conditions were standardized at each research center. Serum concentrations of AFP, AFP‐L3, and PIVKA‐II were measured using the Elecsys (Roche Diagnostics), the Fujirebio assay (Fujirebio Diagnostics), and the ARCHITECT (Abbott Diagnostics), respectively. The technicians performing the laboratory tests were blinded to the diagnosis of the participants. In addition, alkaline phosphatase (ALK) and alanine aminotransferase (ALT) levels were retrieved from electronic medical records.

### Calculation of Published Scores for HCC Detection

The performances of methylation models were compared with those of three published scores for HCC detection, including i) GALAD,^[^ ^22^ ^]^ ii) ASAP,^[^ ^19^ ^]^ and iii) the Doylestown score (age, sex, AFP, alkaline phosphatase, and alanine aminotransferase).^[^ ^21^ ^]^ Sensitivity and specificity were determined at the published cut‐off value (GALAD: −0.63; ASAP: 0.5256; Doylestown: 0.5).^[^ ^19^, ^21^, ^22^ ^]^

### Statistical Analysis

The normalization method of Ct value is described in the section of the COMET assay above. Data were presented with median and interquartile range (continuous variables) or point estimate and 95% CI (sensitivity, specificity, and AUC). The sample size of each statistical test is mentioned in the corresponding figure legend. To assess the between‐group differences, we used i) the Fisher's exact test for categorical variables and ii) the Mann–Whitney test for continuous variables. The AUC was used to estimate the overall performance of cancer detection. The 95% CIs for sensitivity and specificity were generated through the Clopper–Pearson method. Adjusted p values (false discovery rate) for identifying DMRs were calculated using the Benjamini–Hochberg method. The nominal significance level was set as 5%, and all 95% CIs were 2‐sided. All statistical analyses mentioned above were performed using IBM SPSS Statistics 22 or R 4.1.3.

## Conflict of Interest

Yu Xu, Qiancheng You, Lei Zhang, Jiayue Xu, Bingsi Li, Guoqiang Wang, and Shangli Cai are employees of Burning Rock Biotech. No other conflict of interest was reported.

## Author Contributions

T.Y., M.W., N.W., M.P., Y.X., and Q.Y. contributed equally to this work as co‐first authors. T.Y., M.W., M.P., Q.Y., B.L., G.W., S.C., G.L., F.S. performed conceptualization. T.Y., M.W., N.W., M.P., Q.Y., J.X., B.L., S.C., G.L., F.S. performed methodology. All authors performed data curation. T.Y., M.W., N.W., M.P., Q.Y., L.Y., J.X., L.G., X.S., J.X., B.L., G.L., F.S. performed investigation. T.Y., Y.X., Q.Y., L.Z. performed formal analysis. T.Y., Y.X., L.Z. performed visualization. T.Y., Y.X., L.Z. wrote the original draft. All authors wrote, reviewed, and edited the final manuscript. T.Y., B.L., G.W., S.C., G.L., F.S. performed project administration. T.Y., S.C., G.L., F.S. performed supervision.

## Supporting information



Supporting Information

Supplemental Tables

## Data Availability

Research data are not shared.
