# Supplementary material for: Cost‐Effective Identification of Hepatocellular Carcinoma from Cirrhosis or Chronic Hepatitis Virus Infection Using Eight Methylated Plasma DNA Markers
Source: Adv Sci (Weinh). 2025 Mar 26;12(19):2411945. doi: 10.1002/advs.202411945 (PMC12097027; doi:10.1002/advs.202411945)

**Supplemental Materials**

Supplement to: T. Yang, N. Wang, Y. Xu, et al.

Cost-effective identification of hepatocellular carcinoma from cirrhosis or chronic hepatitis virus infection using eight methylated plasma DNA markers:

marker discovery, phase I pilot, and phase II clinical validation

**TABLE OF CONTENTS**

Supplemental Methods 2

Inclusion and Exclusion Criteria 2

Supplemental Figures 3

Supplemental Fig. S1. Methylation levels of the genes associated with the 11 identified MDMs in the TCGA cohort. 3

Supplemental Fig. S2. Methylation levels of the 11 genes across different etiologies in the TCGA cohort. 4

Supplemental Fig. S3. Methylation level of the 11MDMs of the cHCC-CC sample in phase I. 5

Supplemental Fig. S4. Methylation level of the 8 MDMs of the cHCC-CC sample in phase II. 6

Supplemental Fig. S5. Subgroup analysis of sensitivity based on BCLC staging in HCC cases. 7

Supplemental Fig. S6. Comparison of sensitivity among the COMET models, protein markers, and published scores when cut-off values were adjusted to yield a specificity of 97.3%. 8

Supplemental Fig. S7. Comparison of sensitivity among the COMET models, protein markers, and published scores when cut-off values were adjusted to yield a specificity of 94.5%. 9

# Supplemental Methods

## Inclusion and Exclusion Criteria

Inclusion criteria for cancer cases include:

- Age: between 18 and 75;
- Confirmed diagnosis of hepatocellular carcinoma (HCC) or combined HCC-cholangiocarinoma (cHCC-CC);
- Confirmed diagnosis of HBV/HCV chronic infection (HVI) and/or liver cirrhosis (LC);
- Enough pre-cancer treatment plasma samples to ensure cfDNA methylation testing, which were collected within 6 months before diagnosis of HCC or cHCC-CC;

Exclusion criteria for c cancer cases include:

- Participants who were pregnant or lactating;
- Recipient of organ transplant or prior non-autologous (allogeneic) bone marrow or stem cell transplant;
- Recipient of blood transfusion within 7 days prior to blood draw;
- Individuals who received cancer treatment before the blood draw;
- Participants who took other medication with anti-tumor effects within 30 days prior to blood draw;
- Participants with hemorrhagic diseases;
- Participants with autoimmune diseases;
- Participants with concurrent or heterozygous for other malignant tumors or multiple primary tumors;
- Participants who were ultimately diagnosed with non-invasive cancer diseases or other cancers.

Inclusion criteria for HVI/LC controls include:

- Age: between 18 and 75;
- Confirmed diagnosis of HBV/HCV chronic infection (HVI) and/or liver cirrhosis (LC);
- Enough plasma samples to ensure cfDNA methylation testing.

Exclusion criteria for non-cancer participants include:

- Participants who were pregnant or lactating;
- Recipient of organ transplant or prior non-autologous (allogeneic) bone marrow or stem cell transplant;
- Recipient of blood transfusion within 7 days prior to blood draw;
- Participants who took medication with anti-tumor effects within 30 days prior to blood draw;
- Participants with hemorrhagic diseases;
- Participants with autoimmune diseases;
- Participants with current or prior diagnosis of cancer.

# Supplemental Figures

## Supplemental Fig. S1. Methylation levels of the genes associated with the 11 identified MDMs in the TCGA-LIHC cohort.

**
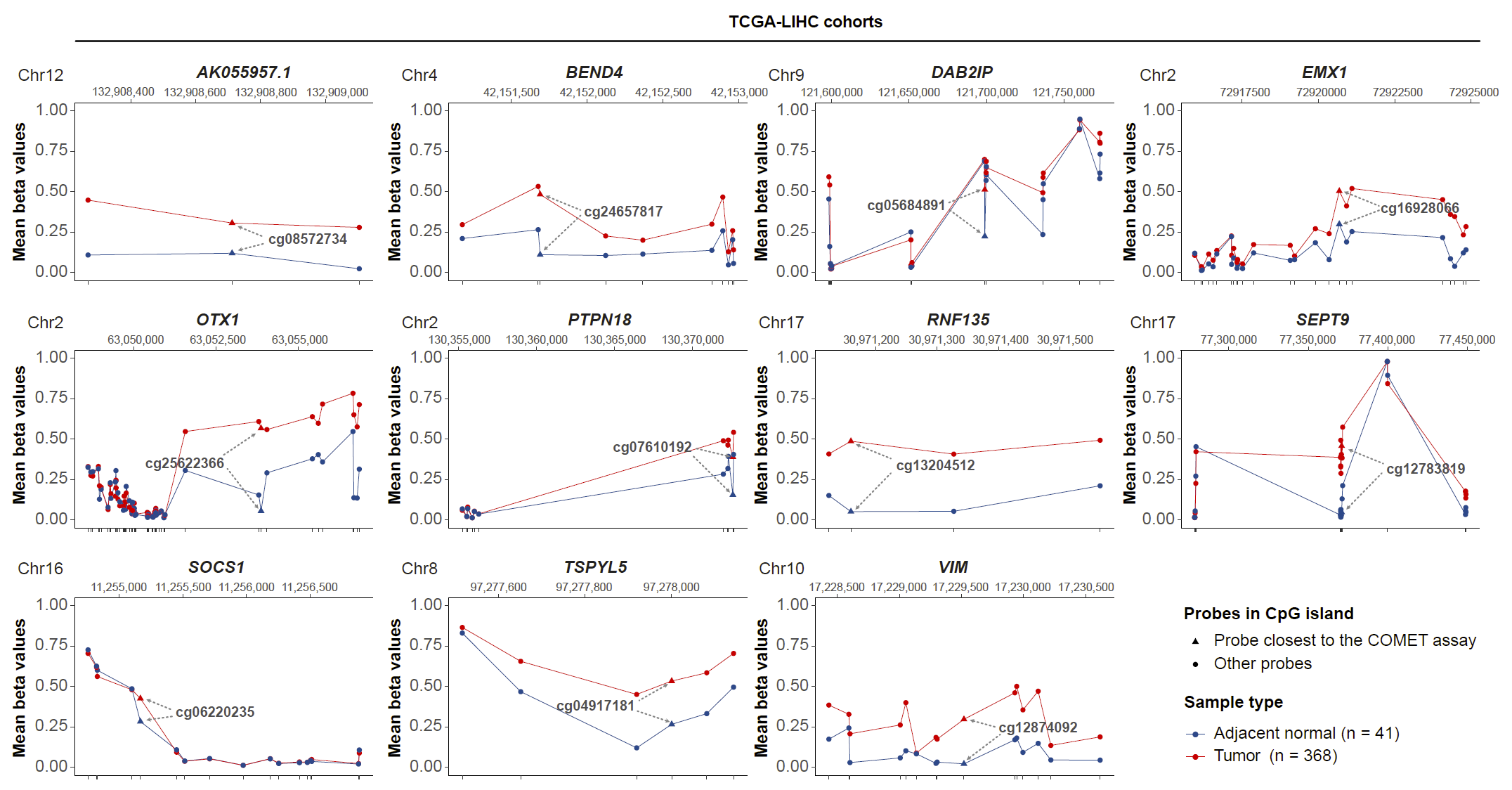
**

## Supplemental Fig. S2. Methylation levels of the 11 genes across different etiologies in the TCGA cohort.

**
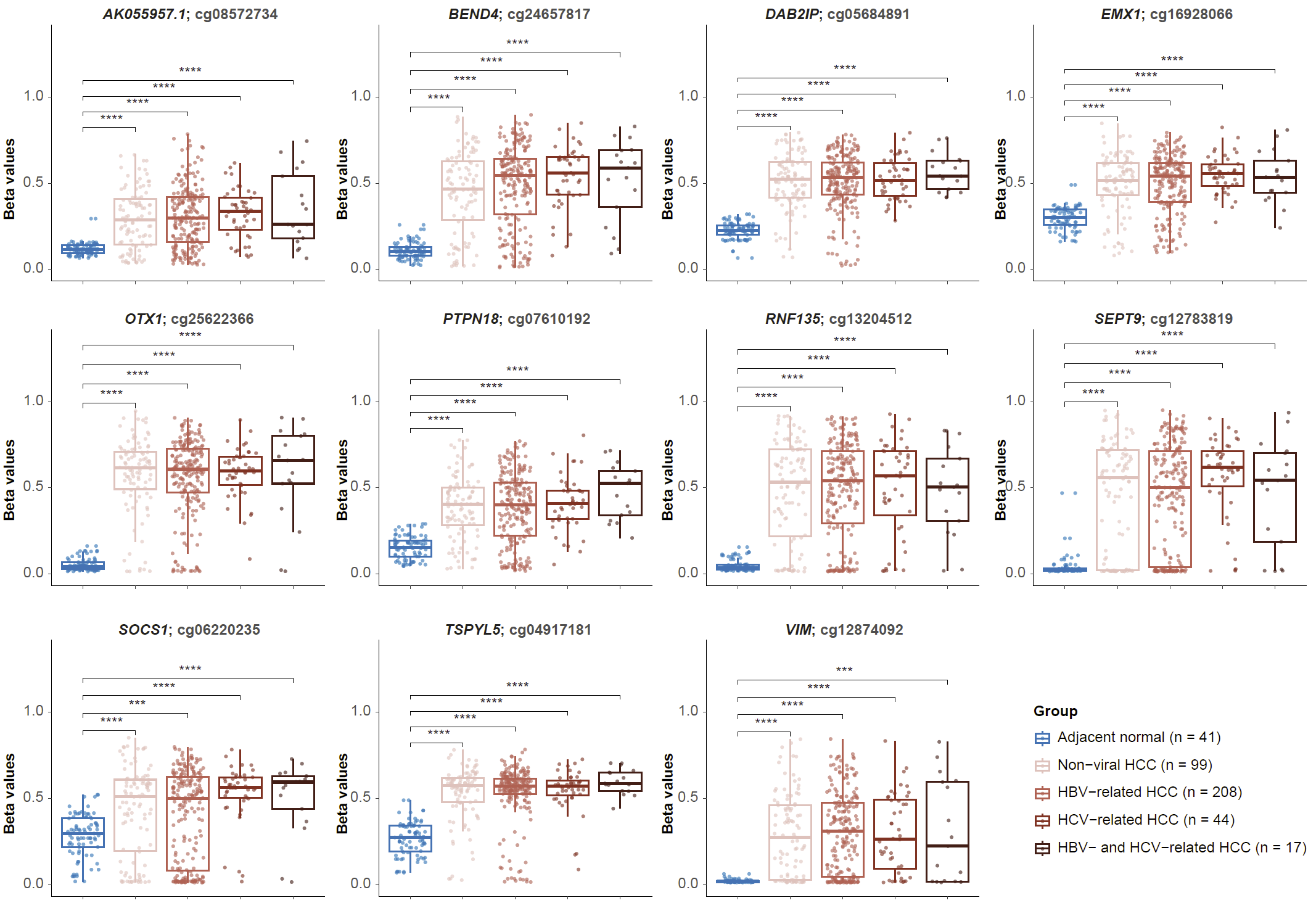
**

## Supplemental Fig. S3. Subgroup analysis of sensitivity based on BCLC staging in HCC cases.


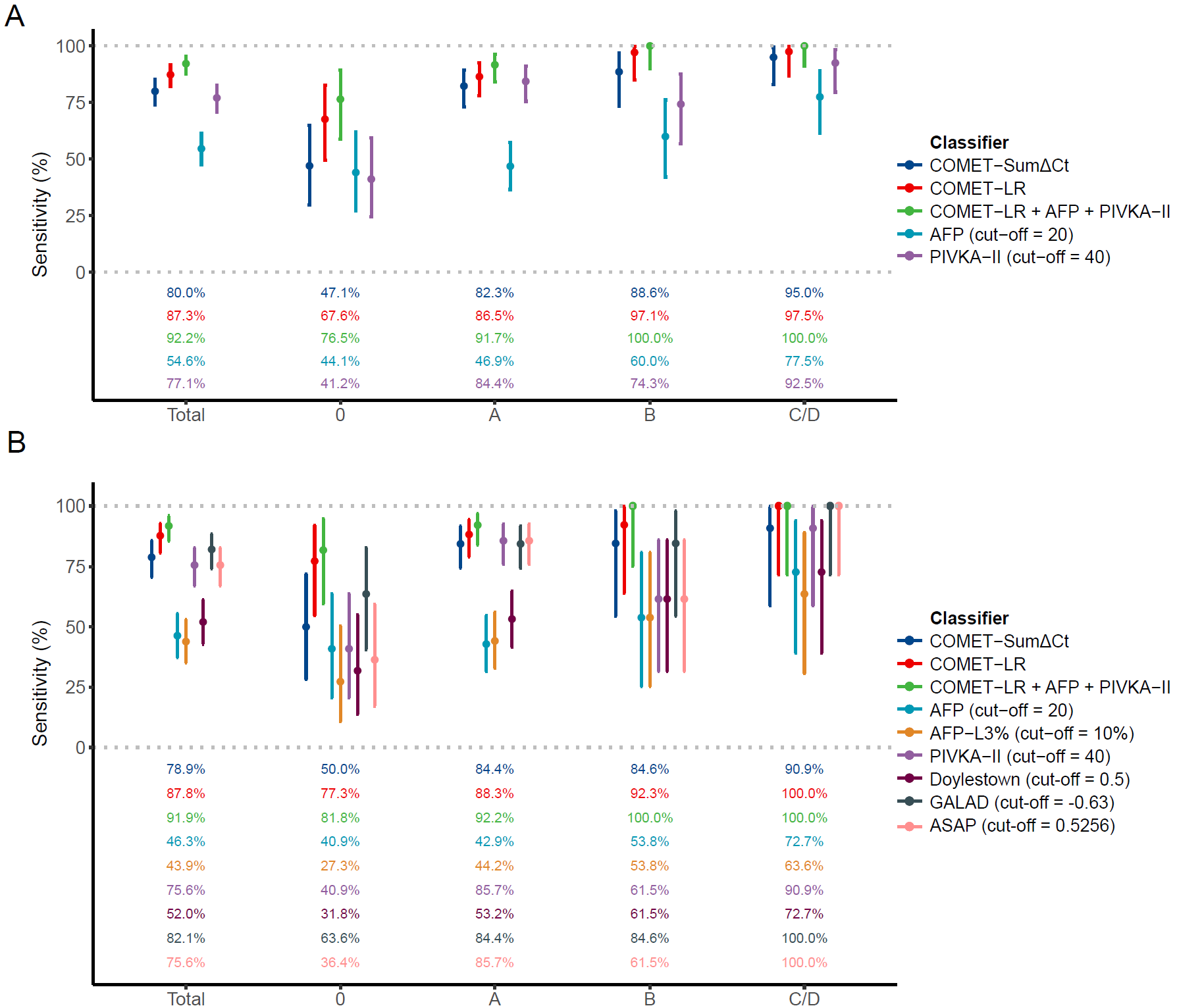


## Supplemental Fig. S4. Comparison of sensitivity among the COMET models, protein markers, and published scores when cut-off values were adjusted to yield a specificity of 97.3%.


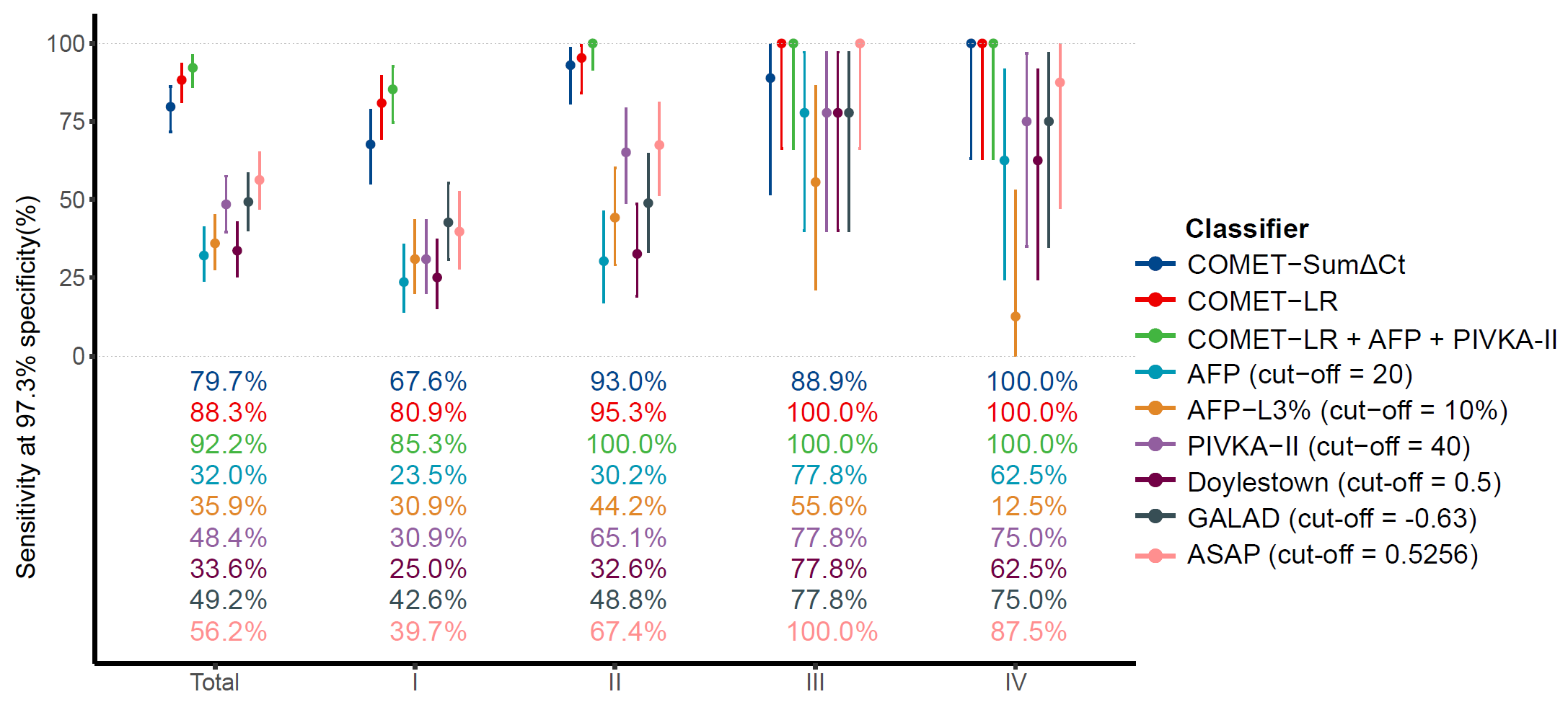


## Supplemental Fig. S5. Comparison of sensitivity among the COMET models, protein markers, and published scores when cut-off values were adjusted to yield a specificity of 94.5%.


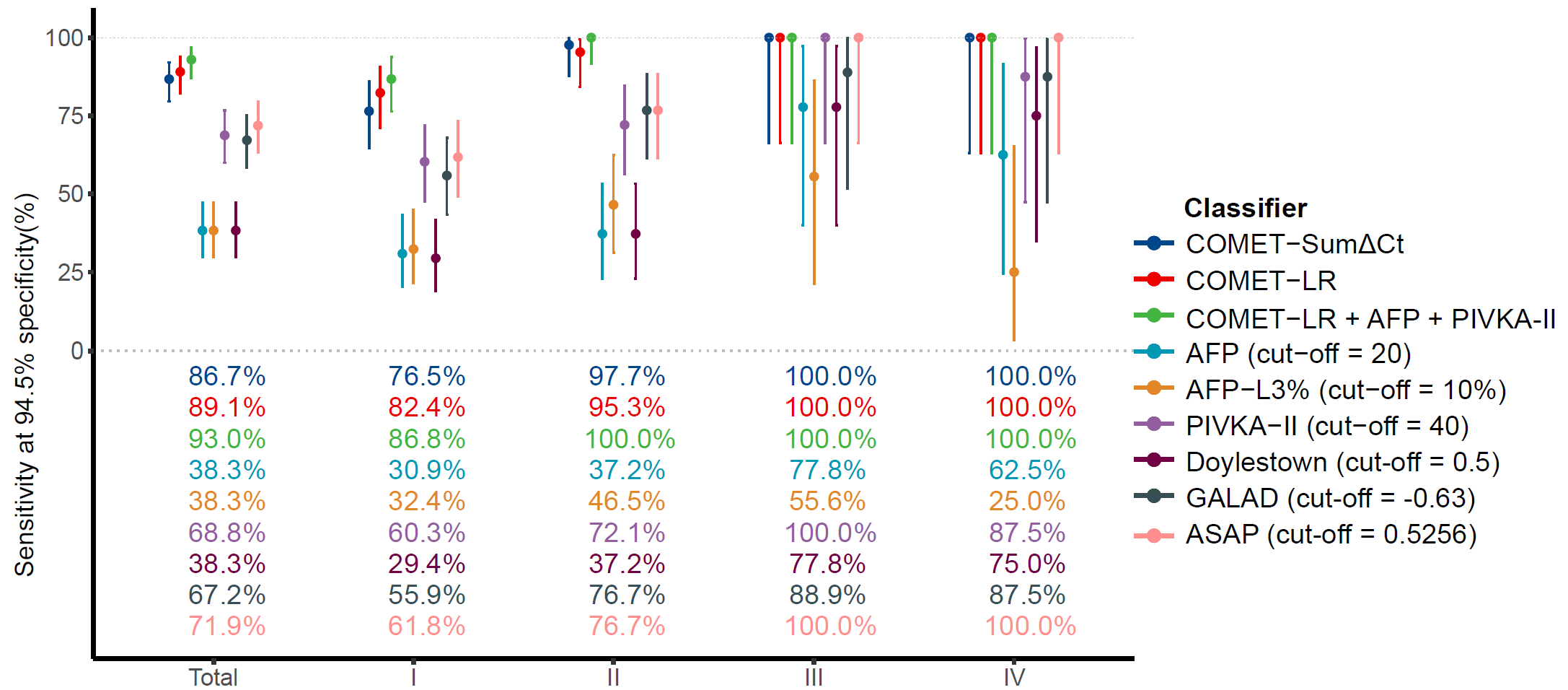


## Supplemental Fig. S6. Methylation levels of the genes associated with the 8 identified MDMs in the ICCs of the TCGA-CHOL cohort.


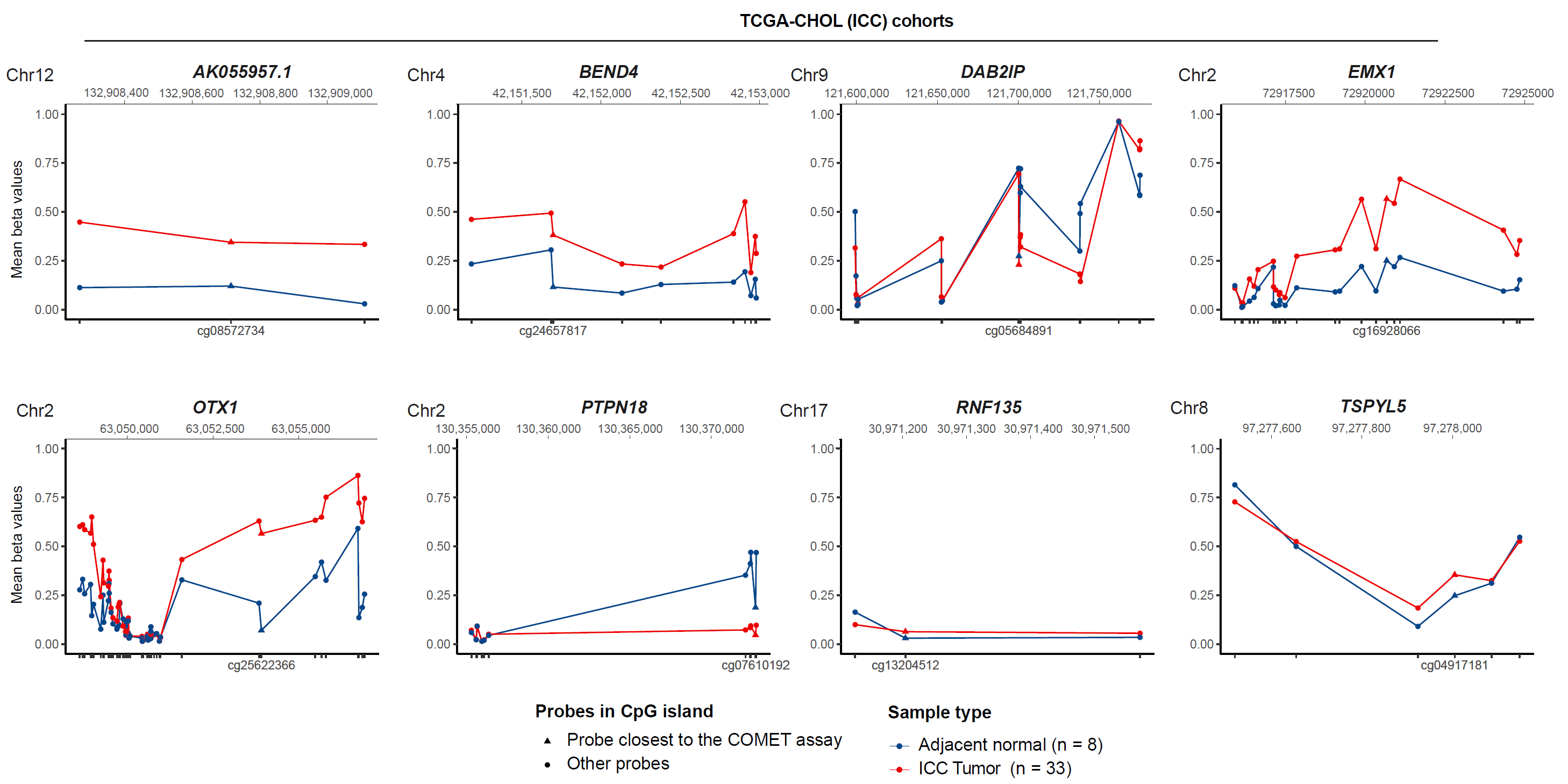


## Supplemental Fig. S7. Methylation levels of the 8 MDMs in controls, HCCs, ICCs, and cHCC-CCs.


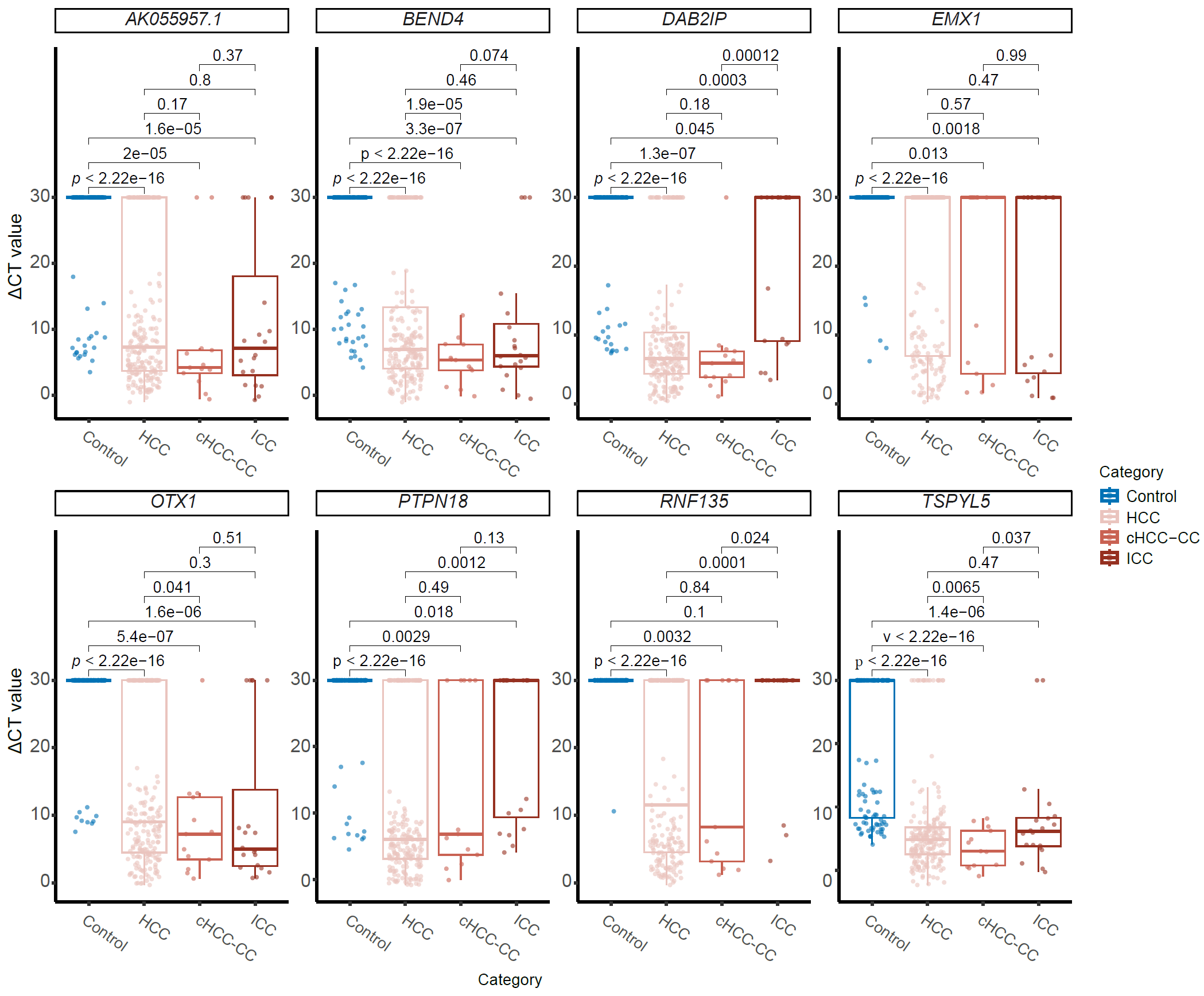


## Supplemental Fig. S8. Sensitivity of methylation models in ICCs and cHCC-CCs.


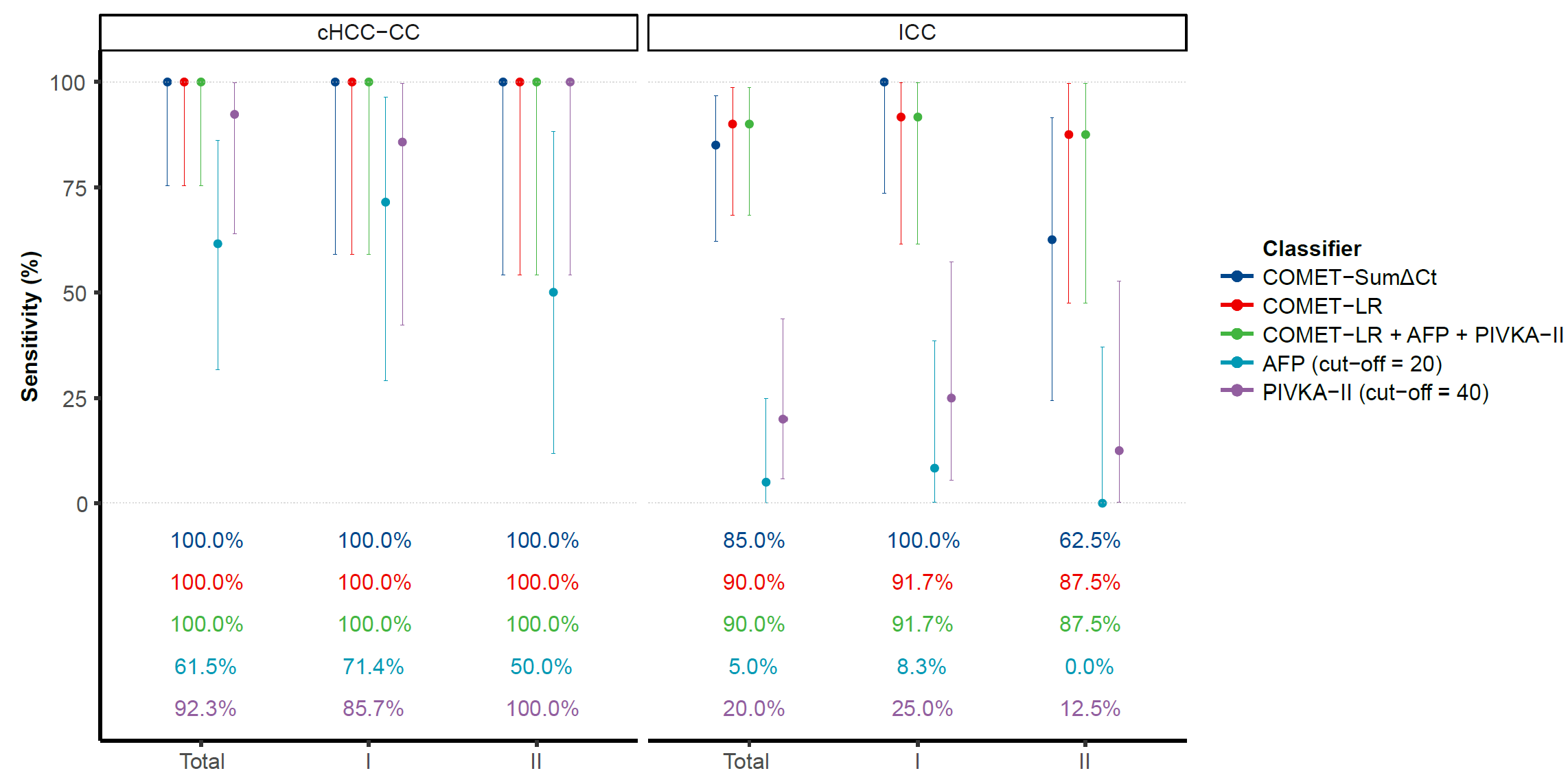

Supplement: Supplementary file 1 — Supporting Information [file ADVS-12-2411945-s002.docx]
